# Influence of biofilm lubricity on shear‐induced transmission of staphylococcal biofilms from stainless steel to silicone rubber

**DOI:** 10.1111/1751-7915.12798

**Published:** 2017-08-03

**Authors:** Niar Gusnaniar, Jelmer Sjollema, Ed D. Jong, Willem Woudstra, Joop de Vries, Titik Nuryastuti, Henny C. van der Mei, Henk J. Busscher

**Affiliations:** ^1^ University of Groningen and University Medical Center Groningen Department of Biomedical Engineering Antonius Deusinglaan 1 9713 AV Groningen The Netherlands; ^2^ Department of Microbiology Universitas Gadjah Mada Yogyakarta Yogyakarta Indonesia

## Abstract

In real‐life situations, bacteria are often transmitted from biofilms growing on donor surfaces to receiver ones. Bacterial transmission is more complex than adhesion, involving bacterial detachment from donor and subsequent adhesion to receiver surfaces. Here, we describe a new device to study shear‐induced bacterial transmission from a (stainless steel) pipe to a (silicone rubber) tube and compare transmission of EPS‐producing and non‐EPS‐producing staphylococci. Transmission of an entire biofilm from the donor to the receiver tube did not occur, indicative of cohesive failure in the biofilm rather than of adhesive failure at the donor‐biofilm interface. Biofilm was gradually transmitted over an increasing length of receiver tube, occurring mostly to the first 50 cm of the receiver tube. Under high‐shearing velocity, transmission of non‐EPS‐producing bacteria to the second half decreased non‐linearly, likely due to rapid thinning of the lowly lubricious biofilm. Oppositely, transmission of EPS‐producing strains to the second tube half was not affected by higher shearing velocity due to the high lubricity and stress relaxation of the EPS‐rich biofilms, ensuring continued contact with the receiver. The non‐linear decrease of ongoing bacterial transmission under high‐shearing velocity is new and of relevance in for instance, high‐speed food slicers and food packaging.

## Introduction

Biofilms are surface‐adhering bacterial communities, in which bacteria have adapted themselves to their adhering state by excretion of extracellular polymeric substances (EPS) providing a protective matrix embedding biofilm inhabitants (Tolker‐Nielsen and Molin, 2000; Høiby *et al*., 2010). Biofilm formation is usually depicted to commence with transport of individual bacteria to a surface where they surface adapt themselves and start growing into a biofilm with emergent properties, amongst which EPS production (Flemming *et al*., 2016). Although convective fluid flow, diffusion and sedimentation are well‐recognized mechanisms of bacterial transport (Picioreanu *et al*., 2000; Wilking *et al*., 2013), in real‐life situations, bacteria are often transmitted from biofilms growing on a donor surface to a receiver one (Hall‐Stoodley and Stoodley, 2005). Bacterial transmission is a much more complex process than bacterial adhesion, as it involves detachment of bacteria from a biofilm on the donor and subsequent adhesion of detached bacteria to the receiver surface where they may grow into a mature biofilm again (Rodríguez *et al*., 2007; Yildiz, 2007).

Bacterial transmission between surfaces is a troublesome, hard to avoid problem especially in hospital environments. Beds, beside tables, carts, bed curtains, bed linen, chairs, closets and floors form a known cause of nosocomial infections, apart from transmission of bacteria from health workers, patients themselves (Zaidi *et al*., 2005; Høiby *et al*., 2011) or medical devices. Intravenous catheters for instance are frequently involved in hospital‐acquired blood stream infections (Moretti *et al*., 2005; Kiertiburanakul *et al*., 2010) in which transmission of endogenous bacteria from the patients or health workers yields bacterial colonization of the catheter (Crnich and Maki, 2002). Bacteria can be transmitted to the catheter surface during its insertion, sliding through hard to disinfect subcutaneous skin layers, while also the use of a guide wire during insertion creates an opportunity for bacteria to enter the catheter hub and lumen (Mermel *et al*., 1991; Crnich and Maki, 2002). Besides being troublesome in hospital environments, bacterial transmission is troublesome in industrial applications, such as food processing (Chaitiemwong *et al*., 2014) and packaging (Arinder *et al*., 2016) but also in ordinary household applications involving toilet surfaces, kitchen sinks, household sponges, floors and carpets (Knox *et al*., 2015).

Bacterial transmission is a multifactorial process governed by transmission time, pressure under which it occurs, types of surfaces involved, the bacterial strain, moisture and temperature (Vickery *et al*., 2004; Vorst *et al*., 2006; Pérez‐Rodríguez *et al*., 2008; Rodriguez *et al*., 2008). Two types of pressures can be identified under which bacterial transmission may take place: compressive and shear pressures. Studies available in the literature on mechanisms of bacterial transmission mostly rely on compressive forces to establish contact between donor and receiver surfaces (Sattar *et al*., 2001; Kusumaningrum *et al*., 2003). However, transmission under shear is at least equally, if not more common, as for instance during slicing of meat (Chaitiemwong *et al*., 2014), intravenous catheter insertion through the skin *(Maki et al.,* 1977) or urinary catheter insertion attracting periurethral bacteria to the catheter surface (Nicolle, 2014). Whereas bacterial transmission is more complicated than adhesion, bacterial transmission under shear is more complicated than transmission under compression. Transmission under shear adds the shearing velocity as another important parameter to the process and therewith friction of the biofilm against the receiver surface and of internal layers within the biofilm against each other.

Despite its practical relevance, a device that allows to study bacterial transmission under controlled shear conditions is not available. Therefore, we here present a new device to study biofilm transmission from the lumen of cylindrically shaped stainless steel donors to the extraluminal surface of a silicone rubber receiver tube under shear. Transmission will be studied for two different velocities at which donor and receiver surfaces are sheared against each other with a biofilm in between. To assess the influence of EPS on transmission, two staphylococcal species, each represented by two different strains were used as follows: two EPS‐producing strains (*Staphylococcus epidermidis* ATCC 35984 and *Staphylococcus aureus* ATCC 12600) and two non‐EPS‐producing strains (*S. epidermidis* 252 and *S. aureus* 5298). As EPS may have a large impact on the lubricity of a biofilm, colloidal probe atomic force microscopy (AFM) was applied to compare the lubricity of biofilms formed by the EPS‐ and non‐EPS‐producing strains. Both *S*. *epidermidis* and *S. aureus* are common pathogens in intravascular catheter‐associated blood stream (Mermel *et al*., 2009) and other nosocomial infections (Otto, 2009).

## Results

**Figure 1:**
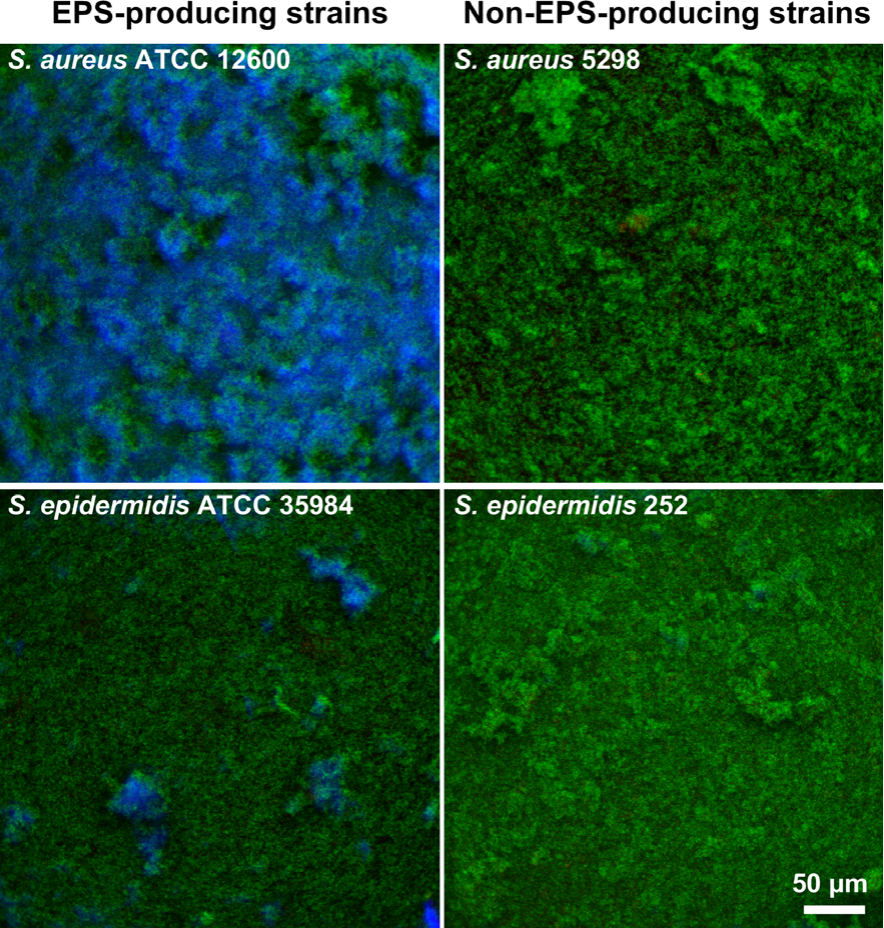
Confocal laser scanning microscopy images of biofilms on stainless steel of EPS‐ and non‐EPS‐producing staphylococcal strains, stained with LIVE/DEAD stain and Calcofluor white (blue fluorescence) to demonstrate EPS production.

Confocal laser scanning microscopy (CLSM) images of the staphylococcal biofilms on stainless steel plates clearly showed blue‐fluorescent patches indicative of the presence of EPS in the EPS‐producing strains, that were absent in the non‐producing strains (Fig. 1). Note that the strong EPS producer, *S. aureus* ATCC12600, clearly shows more blue‐fluorescent patches than the moderate EPS producer, *S. epidermidis* ATCC 35984.

**Figure 2:**
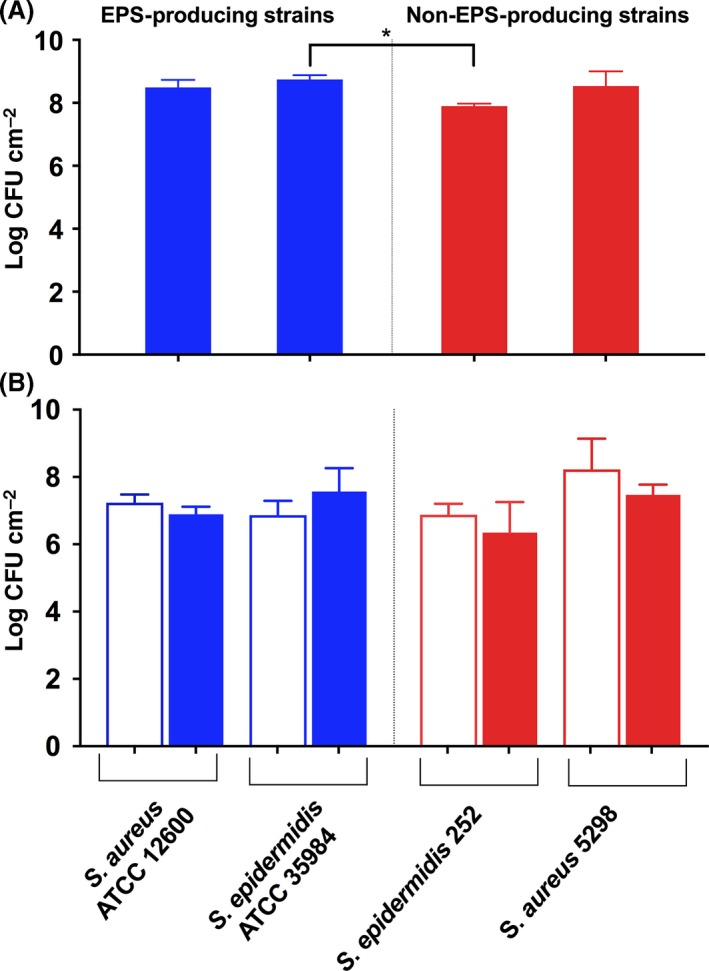
Numbers of staphylococci in biofilms grown on stainless steel donors before transmission and left‐behind after transmission for EPS‐producing and non‐EPS‐producing strains. A. Number of staphylococci in biofilms before transmission. B. Number of staphylococci in biofilms left‐behind after transmission. Open bars represent bacterial numbers after transmission at a low‐shearing velocity, whereas closed bars represent bacterial numbers after transmission at high velocity. Error bars indicate the standard deviations over triplicate experiments with biofilms grown from different cultures. Significant differences at *P *<* *0.05 between strains are indicated by an asterisk**.**

Figure 2A indicates that the number of staphylococci in biofilms on the luminal sides of the stainless steel pipes before transmission equalled around 10^8^ CFU cm^−2^, although the number of *S. epidermidis* 252 was slightly lower than of *S. epidermidis* ATCC 35984 (*P *<* *0.05; one‐way ANOVA followed by Dunnett's multiple comparisons test). The number of staphylococci left on the luminal sides of the stainless steel pipes after transmission amounted around 1.5 × 10^7^ CFU cm^−2^ (Fig. 2B), with no significant differences between numbers of bacteria of the same strain after transmission at low or high‐shearing velocities (Kruskal–Wallis followed by Dunn's multiple comparison test).

**Figure 3:**
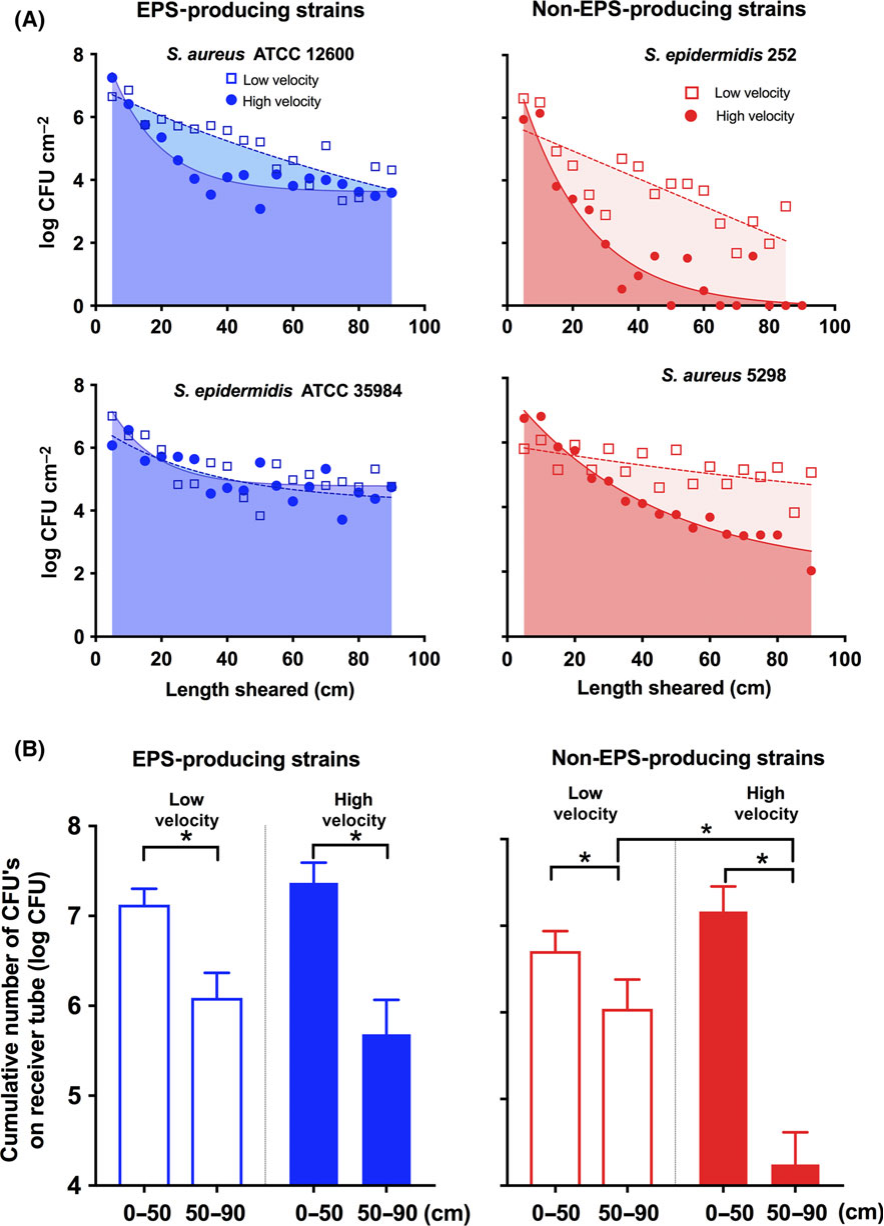
A. The number of staphylococcal CFUs on silicone rubber receiver surfaces per unit area transmitted from a biofilm on the luminal side of a stainless steel pipe as a function of the tube length sheared by the donor pipe (see also Fig. 5D) for EPS‐ (left panel) and non‐EPS‐producing (right panel) strains. The stainless steel pipe was drawn over the silicone rubber tube at low (open symbols, shaded region) or high (closed symbols, fully coloured region) shearing velocity. Drawn lines represent an exponential fit to the transmission data under high‐shearing velocity, while transmission under low velocity is fitted to a linear function (dotted lines). Error bars indicate the standard deviations over triplicate experiments with biofilms grown from different cultures. B. Averaged cumulative numbers of staphylococcal CFUs for both EPS‐producing and non‐EPS‐producing staphylococcal strains on the first (0–50 cm) and second (50–90 cm) parts of the silicone rubber receiver tubes transmitted from biofilms on stainless steel donor pipes under low‐ and high‐shearing velocities. Error bars indicate the standard deviations over triplicate experiments with biofilms grown from different cultures. Significant differences at *P *<* *0.05 between strains are indicated by an asterisk.

Figure 3A shows the numbers of bacteria per unit area of the silicone rubber tube transmitted by drawing the stainless steel pipe over the tube as a function of the length of the tube sheared by the stainless steel pipe. For the low‐shearing velocity, the log‐numbers of adhering bacteria decreased almost linearly over the entire 90 cm length of silicone rubber tube sheared for the EPS‐producing and non‐EPS‐producing strains (Fig. 3A). In addition, there were no significant (Kruskal–Wallis followed by Dunn's multiple comparison test) differences in the area under the curves between the strains, representing the cumulative number of bacteria transmitted over the entire 90 cm length of the silicone rubber tube (note this may not be directly trivial from the graphs due to the use of a logarithmic *Y*‐axis). At the high‐shearing velocity, the decrease in bacterial numbers transmitted was initially faster than at low velocity, but levelled off to a constant transmission of bacteria from the donor to the receiver (see also Fig. 3A).

The highest cumulative numbers of staphylococci were transmitted to the first 50 cm of tube length, but without significant differences between EPS‐producing and non‐EPS‐producing strains and regardless of the shearing velocity (see Fig. 3B). Differences in transmission between the two different types of strains and the two shearing velocities applied became evident only over the second half of the silicone rubber tube (50–90 cm). Although the transmission of EPS‐producing strains to the second part of the tube was not affected by the shearing velocity, non‐EPS‐producing strains were transmitted in significantly (*P *<* *0.05, Mann–Whitney test) lower cumulative numbers to the second half of the tube length at the high‐shearing velocity than at the low‐shearing velocity.

**Figure 4:**
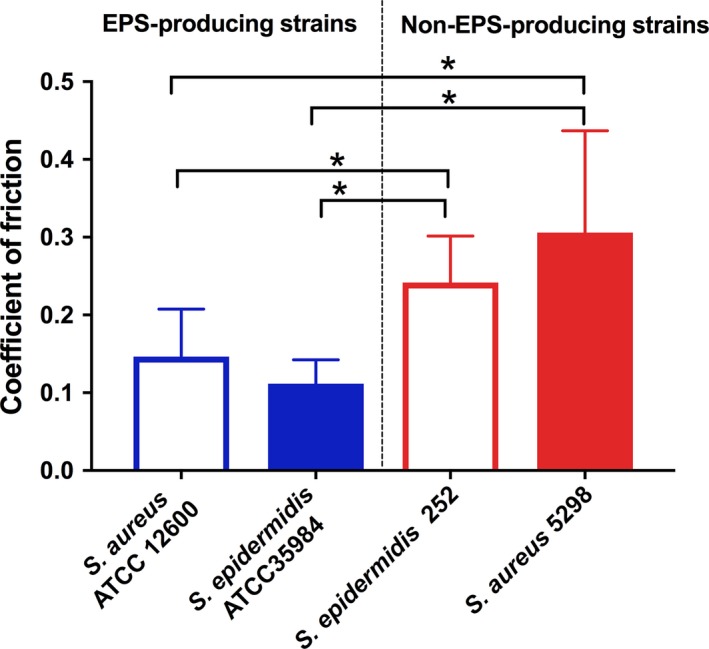
Coefficients of friction of staphylococcal biofilms of EPS‐producing and non‐EPS‐producing strains on stainless steel, obtained using lateral probe atomic force microscopy. Error bars indicate the standard deviations over triplicate experiments with biofilms grown from different cultures. Significant differences at *P *<* *0.05 (Mann–Whitney non‐parametric test) between strains are indicated by an asterisk.

Staphylococcal biofilms of EPS‐producing strains as compared with non‐EPS‐producing strains (Fig. 4) decreased the coefficient of friction significant (*P *<* *0.05, Mann–Whitney test).

## Discussion

We have designed a simple device to study shear‐induced bacterial transmission from the inside of a pipe to the extraluminal side of a tube at different shearing velocities. To demonstrate the possibilities of our design, we used a stainless steel pipe and a silicone rubber tube in combination with biofilms grown by EPS and non‐EPS‐producing staphylococcal strains. Usually, transmission data in the literature are highly irreproducible as transmission is made up of adhesion and detachment, which are already two phenomena that can be measured with relatively low experimental reproducibilities of only 30% on average (Heydorn *et al*., 2000; Lewandowski *et al*., 2004; Lüdecke *et al*., 2014). Our transmission device yields data with a reproducibility, that is comparable with other experimental methods involving either bacterial adhesion or detachment (Jun and Xu, 2014; Yoda *et al*., 2014). Considering that both adhesion and detachment make up a transmission experiment, the accuracy that our design can achieve for transmission data is therefore remarkable. This accuracy is mainly achieved by consecutive transmission measurements using the same donor surface, therewith balancing out inaccuracies in bacterial enumeration. A similar method with consecutive transmissions was described recently for transmission and decontamination of *S. aureus* from hand to contact surfaces and vice versa (Arinder *et al*., 2016), albeit here transmission was established by compression. Shear‐induced transmission experiments were described earlier for bacterial transmission from rotary slicers to meat products, but shearing velocity could not accounted for as it varied over the radius of the rotary slicer (Vorst *et al*., 2006; Chaitiemwong *et al*., 2014).

In our device, we controlled the shearing velocity during transmission by adjusting the velocity at which the pipe was pulled over the tube. Neither at low nor high‐shearing velocities did we see cumulative transmission of a biofilm as a whole from the more hydrophilic, donor stainless steel to the more hydrophobic, receiver silicone rubber surface over the 90 cm length of tube sheared (compare Figs 2 and 3B). Instead, biofilm was gradually transmitted from the donor surface upon shearing over an increasing length of receiver tube (see Fig. 3A). This is despite the fact, that bacterial adhesion forces towards hydrophobic surfaces are generally stronger than to hydrophilic surfaces (Fletcher and Loeb, 1979; Donlan, 2002). Therefore, it must be concluded that transmission occurs through cohesive failure in the biofilm mass and not through adhesive failure at the interface between the stainless steel donor and the staphylococcal biofilm.

There are interesting differences in the way shearing velocity and ability of the strain to produce EPS affect transmission. The amount of biofilm transmitted from highly lubricious biofilm of EPS‐producing staphylococci along the length of the silicone rubber tube is not significantly different when sheared at low or high velocity (Fig. 3A) and accordingly there is no significant difference in the cumulative amount of biofilm transmitted at the two velocities (Fig. 3B). Importantly, as a result of the substantial numbers of bacteria left in the pipe after transmission (Fig. 2B), transmission of staphylococci from the donor to the receiver continues after 90 cm has been drawn over by the stainless steel pipe. Velocity is probably not influential, because biofilms of EPS‐producing staphylococci are highly lubricious (Fig. 4). Moreover, EPS in the biofilm matrix will contribute to the viscoelasticity of a biofilm (Klapper *et al*., 2002; Stewart *et al*., 2015). This allows fast relaxation (Peterson *et al*., 2015) of the biofilm thickness after transmission from the donor to the receiver in order to maintain contact between the biofilm and the shearing receiver surface.

Stress relaxation is slower for biofilms with less EPS (Peterson *et al*., 2013), while in addition biofilms of non‐EPS‐producing strains are less lubricious (Fig. 4). As a result, their higher coefficient of friction will cause a rapid decrease in staphylococcal transmission along the length of tube, yielding a gap between biofilm remaining on the donor and the receiver tube due to rapid thinning of the less lubricious biofilms under high‐shearing velocities. At the high velocity, that is high shear forces, this gap may more readily develop leading to a low‐level transmission of staphylococci from the donor surface, especially as biofilms of non‐EPS‐producing strains cannot fill this gap through viscoelastic relaxation within the time frame shearing within the pipe (Peterson *et al*., 2013).

Transmission phenomena have not been studied very often ((Tacconelli *et al*., 2014), and the current study adds important new aspects. Earlier studies have indicated a linear decrease in log‐numbers of transmitted bacteria upon ongoing transmission (Vorst *et al*., 2006; Chaitiemwong *et al*., 2014; Arinder *et al*., 2016) but did not include the impact of shearing velocity. In our study, especially a high‐shearing velocity makes ongoing transmission less efficacious in a non‐linear way. Moreover, the present study reveals that biofilms of EPS‐ and non‐EPS‐producing strains behave differently with respect to shear‐induced transmission.

## Conclusions

Staphylococcal biofilm transmission under shear from a stainless steel to a silicone rubber surface occurs through cohesive failure in the biofilm and not through failure at the interface between the donor surface and the biofilm. At high‐shearing velocities, more lubricious, EPS‐rich staphylococcal biofilms transmit bacteria over longer distances to the receiver tube than non‐EPS‐producing ones, while less bacteria were transmitted over longer distances from non‐EPS‐producing strains. Thus whereas earlier transmission studies have indicated a linear decrease in log‐numbers of transmitted bacteria upon ongoing transmission, we conclude that high‐shearing velocities make ongoing transmission less efficacious in a non‐linear way.

## Experimental procedures

### Bacterial strains and harvesting

Two staphylococcal species, each including two EPS‐producing (*S. epidermidis* ATCC 35984 (a moderate slime producer) and *S*. *aureus* ATCC 12600 (a strong slime producer)) and two non‐EPS‐producing (*S. epidermidis* 252 and *S. aureus* 5298) strains, were used in this study. The strains were taken from frozen stocks and incubated on blood agar plates at 37°C for 24 h. A single colony was taken, precultured in 10 ml of tryptone soya broth (TSB; Oxoid Ltd, Basingstoke, England) at 37°C for 24 h and subsequently used to inoculate a main culture of 200 ml TSB. Bacteria were harvested by centrifugation (3 × 5 min, 5000 g, 10°C) and washed twice with sterile phosphate‐buffered saline (PBS; 10 mM potassium phosphate, 0.15 M NaCl, pH 7). To break bacterial aggregates, staphylococci were sonicated for 3 × 10 s and re‐suspended in PBS to a density of 1 × 10^9^ bacteria ml^−1^, as determined using a Bürker‐Türk counting chamber.

### Preparation of surfaces

#### Stainless steel pipes

Stainless steel 304 pipes (1 cm length, 4.00 mm inner diameter) were used. Pipes were cleaned by sonication for 5 min with 2% RBS 35 detergent (Omnilabo international BV, Breda, the Netherlands), rinsed with demineralized water, washed in methanol and rinsed with demineralized water prior to autoclaving. The luminal sides of the sterilized pipes were used to grow biofilms on and act as donor surfaces during transmission experiments. Similar plate material was cleaned as described above and cut to samples of 1.5 × 1.5 cm. Plate material thus prepared had a water contact angle of 35 degrees, classifying it as moderately hydrophilic.

#### Silicone rubber tubes

Silicone rubber (Versitec; Rubber BV, Hilversum, the Netherlands) tubes (90 cm length, 3.93 mm outer diameter) were cut into nine segments of 10 cm length. Tube segments were cleaned by sonication for 5 min with 2% RBS 35 detergent, rinsed with sterile demineralized water, immersed in 70% ethanol for 5 min and finally rinsed with sterile demineralized water. The sterilized extraluminal surfaces of the tube segments were used as a receiver material during transmission experiments. Silicone rubber is a hydrophobic material with a water contact angle of 108 degrees.

### Biofilm growth

The stainless steel pipes were placed in 12‐well tissue culture plates, containing 4 ml of a staphylococcal suspension to allow bacteria to adhere for 1 h at room temperature under rotary‐shaking (100 rpm). Subsequently, each pipe was transferred into another 12‐well tissue culture plate, containing 4 ml of fresh TSB. Staphylococci were grown aerobically at 37°C in a rotating shaker (100 rpm) for 72 h to form a biofilm. Pipes were transferred aseptically into a new 12‐well tissue culture plate containing 4 ml of fresh TSB every 24 h. Staphylococcal biofilms were grown similarly on stainless steel plate material for measurement of biofilm lubricity and EPS production demonstrated using CLSM.

To this end, biofilm‐covered samples were immersed in LIVE/DEAD stain (BacLight™, Molecular probes, Leiden, the Netherlands) containing SYTO9 (3.34 mM) and propidium iodide (20 mM) mixed with Calcofluor White (8 mM Fluorescent Brightener 28, Sigma‐Aldrich, St Louis, MO, USA) for 30 min, while being kept in the dark at room temperature. LIVE/DEAD staining allowed assessment of live (green fluorescent) and dead bacteria (red fluorescent) in a biofilm. Calcofluor White is a polysaccharide staining agent used to visualize EPS (Stewart *et al*., 2009). After washing with phosphate‐buffered saline (PBS, 10 mM potassium phosphate and 150 mM NaCl, pH 7.0),biofilm‐covered samples, immersed in PBS, were imaged using a CLSM (Leica TCS‐SP2, Leica Microsystems Heidelberg GmbH, Heidelberg, Germany) at 40× magnification with laser excitation at 488 nm and 543 nm for LIVE/DEAD stain and 405 nm for Calcofluor White. Images were stacked, optimized and analysed using fiji software (Schindelin *et al*., 2012).

### Biofilm transmission

**Figure 5:**
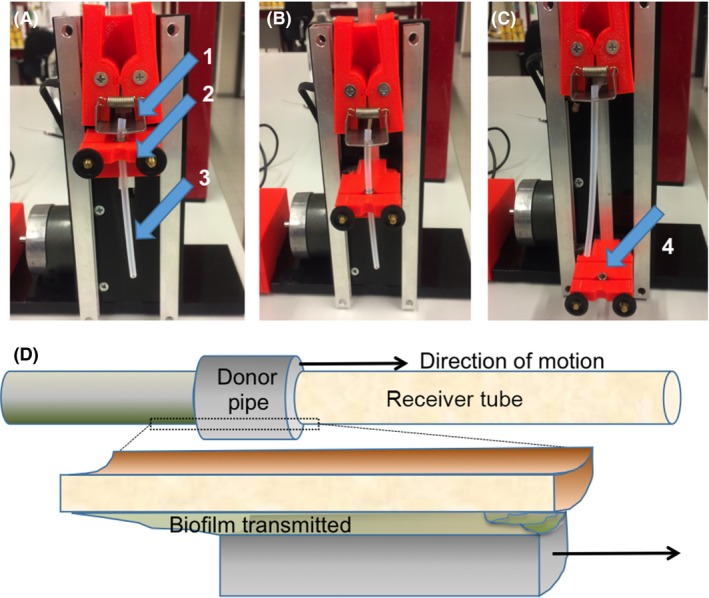
Home‐made device to study bacterial transmission under shear. A. The first centimetre of a 9 cm long silicone rubber tube segment (3) is clamped into a tube holder (1). B. The pipe holder (2) with the pipe inserted is subsequently pulled downward at a defined shearing velocity over the remaining length of the tube. C. Once the pipe is pulled over the entire tube, the silicone rubber tube is taken out of the device for enumeration of the number of bacteria transmitted. Note the stainless steel pipe (4) is now visible. D. Schematics showing a compressed biofilm in between the shearing pipe and tube.

Biofilm transmission was studied in a home‐made device (see Fig. 5), designed to control the velocity at which donor and receiver surfaces were sheared. First, the stainless steel pipe was clamped into the pipe holder (see Fig. 5A), and the first 1.5 cm of a tube segment was manually pulled through the pipe and subsequently clamped in the tube holder (see also Fig. 5A). Next, while keeping the tube at a fixed position, the pipe holder was pulled downwards over the remaining 8.5 cm length of the tube segment (Fig. 5B and C). This procedure was repeated for the other eight tube segments, with roughly 1 min time interval in between due to replacing of the previous segment. Using this procedure, a total length of 90 cm silicone rubber tube was sheared against 1 cm length of biofilm grown inside the stainless steel pipe. For practical reasons, it was impossible to use one length of 90 cm. The pipe holder was pulled over the silicone rubber tube at two velocities of 1 cm s^−1^ (designated as ‘low’) and 10 cm s^−1^ (‘high’). A schematic outline of the method is presented in Fig. 5D.

After transmission, each tube segment was cut into three sections with lengths of 1.5 cm (the clamped section), 3.5 cm and 5.0 cm. The last two sections were used for enumeration of the number of bacteria on the tube. The clamped section was discarded for further analysis.

### Number of bacteria in biofilms prior to and after transmission

To determine the numbers of staphylococci in biofilms inside the stainless steel pipe before and after transmission, it was first ascertained that the outside of the pipes was devoid of adhering bacteria by wiping with cotton tipped applicators (Raucotupf, Lohmann & Rauscher, Germany). Following cleaning of the outside, the lumen of a pipe was brushed using 5 mm interdental brushes (Albert Heijn, Zaandam, the Netherlands) in 5 ml of PBS, after which the brush and the pipe were vortexed and subsequently sonicated for 3 × 10 s to break bacterial aggregates, and the sonicate was serially diluted. Twenty μl bacterial suspension droplets were put on tryptone soya agar (TSA), plates were incubated at 37°C for 24 h and the number of colony‐forming units (CFUs) were counted. The numbers of CFUs on the extraluminal side of the silicone rubber tube sections were determined analogously and summed to yield the cumulative amount of biofilm transmitted over the first 50 cm and last 40 cm of the silicone rubber tube. The numbers of CFUs on the discarded clamped sections were assumed to equal the average of the neighbouring sections. All experiments were carried out in triplicate with staphylococcal biofilms grown from different cultures.

### Lubricity of EPS‐producing and non‐EPS‐producing staphylococcal biofilms

Biofilm lubricity was assessed by their coefficient of friction. Coefficients of friction towards a colloidal AFM probe (for details, see Ralston *et al*., 2005) on EPS‐producing and non‐EPS‐producing staphylococcal biofilms were measured with an AFM (Nanoscope IV Dimension™ 3100) equipped with a Dimension Hybrid XYZ SPM scanner head (Bruker, New York‐USA). Rectangular, tipless cantilevers were calibrated for their exact torsional and normal stiffness using afm tune it v2.5 software (Green *et al*., 2004). Subsequently, a 22 μm silica sphere (Microparticles GmbH, Berlin, Germany) was glued to a cantilever with an epoxy glue (Pattex, Brussels, Belgium) using a micromanipulator (Narishige group, Tokyo, Japan). The colloidal probe was incrementally loaded and unloaded in steps of 5 nN, up to a maximal normal force of 50 nN. Lateral deflection was observed at a scanning angle of 90 degrees at a velocity of 15 μm s^−1^, converted into friction force and plotted against the normal forces applied yielding coefficients of friction by linear least‐squares fitting. Friction analyses were performed in triplicate for each strain with separately grown biofilms.

### Statistical analyses

Statistics were performed using graphpad prism (GraphPad Software, La Jolla, CA, USA). All experimental data were presented as means ± standard deviations over triplicate experiments. Differences were analysed using One‐Way ANOVA or as indicated and considered to be statistically significant when *P *<* *0.05.

## Conflict of interest

None declared.
